# Design of Adaptive-Robust Controller for Multi-State Synchronization of Chaotic Systems with Unknown and Time-Varying Delays and Its Application in Secure Communication

**DOI:** 10.3390/s21010254

**Published:** 2021-01-02

**Authors:** Ali Akbar Kekha Javan, Afshin Shoeibi, Assef Zare, Navid Hosseini Izadi, Mahboobeh Jafari, Roohallah Alizadehsani, Parisa Moridian, Amir Mosavi, U. Rajendra Acharya, Saeid Nahavandi

**Affiliations:** 1 Faculty of Electrical Engineering, Gonabad Branch, Islamic Azad University, Gonabad 6518115743, Iran; kikha_akbar@yahoo.com; 2 Biomedical Data Acquisition Lab (BDAL), Faculty of Electrical Engineering, K. N. Toosi University of Technology, Tehran 1996715433, Iran; afshin.shoeibi@gmail.com; 3 Computer Engineering Department, DDEMS Lab, Ferdowsi University of Mashhad, Mashhad 9177948974, Iran; 4 Department of Electrical and Computer Engineering, Isfahan University of Technology, Isfahan 8415683111, Iran; hnavidh@yahoo.com; 5 Electrical and Computer Engineering Faculty, Semnan University, Semnan 3513119111, Iran; mahbube.jafari@yahoo.com; 6 Institute for Intelligent Systems Research and Innovation (IISRI), Deakin University, Waurn Ponds, VIC 3217, Australia; ralizadehsani@deakin.edu.au (R.A.); saeid.nahavandi@deakin.edu.au (S.N.); 7 Faculty of Engineering, Science and Research Branch, Islamic Azad University, Tehran 1477893855, Iran; parisamoridian@yahoo.com; 8 Faculty of Civil Engineering, Technische Universität Dresden, 01069 Dresden, Germany; 9 John von Neumann Faculty of Informatics, Obuda University, 1034 Budapest, Hungary; 10 School of Economics and Business, Norwegian University of Life Sciences, 1430 Ås, Norway; 11 Department of Biomedical Engineering, School of Science and Technology, Singapore University of Social Sciences, Singapore 599494, Singapore; aru@np.edu.sg; 12 Department of Electronics and Computer Engineering, Ngee Ann Polytechnic, Singapore 599489, Singapore; 13 Department of Bioinformatics and Medical Engineering, Asia University, Taichung City 41354, Taiwan

**Keywords:** time-delayed chaotic systems, adaptive-robust control, multi-state synchronization, unknown time delays, secure communications, time varying parameter, circular multi state synchronization

## Abstract

In this paper, the multi-state synchronization of chaotic systems with non-identical, unknown, and time-varying delay in the presence of external perturbations and parametric uncertainties was studied. The presence of unknown delays, unknown bounds of disturbance and uncertainty, as well as changes in system parameters complicate the determination of control function and synchronization. During a synchronization scheme using a robust-adaptive control procedure with the help of the Lyapunov stability theorem, the errors converged to zero, and the updating rules were set to estimate the system parameters and delays. To investigate the performance of the proposed design, simulations have been carried out on two Chen hyper-chaotic systems as the slave and one Chua hyper-chaotic system as the master. Our results showed that the proposed controller outperformed the state-of-the-art techniques in terms of convergence speed of synchronization, parameter estimation, and delay estimation processes. The parameters and time delays were achieved with appropriate approximation. Finally, secure communication was realized with a chaotic masking method, and our results revealed the effectiveness of the proposed method in secure telecommunications.

## 1. Introduction

Applications of digital telecommunications can be seen in all aspects of daily life and all industries including medicine and education, as well as social interactions [1]. Hence, there is a need to have secure communication. To do this, many cryptography methods have been introduced, mainly encoding the data from the sender and decoding them at the receiver [1]. Cryptographical methods are based on various schemes, and a number of related papers have been published in this field [2,3,4,5]. There has been a considerable amount of work done in this field, and chaotic systems play a significant role among all cryptographical schemes [6,7,8].

One of the fundamental characteristics of chaotic systems is the extreme response to small changes in initial circumstances. The main goal of proposing synchronization methods is the suitable tuning of controller parameters in chaotic systems [9,10,11,12]. In chaotic synchronization, the vector state of the master system follows the slave system [13]. Various control-based schemes have been used previously for synchronization, such as adaptive [14,15,16], sliding mode [17,18], back stepping [19], fuzzy [20], predictive [21], and robust [22].

The work on applications of chaotic synchronization in secure communication has grown dramatically in recent years [23,24,25,26,27,28,29,30,31,32,33,34,35,36]. Developing control systems for synchronization of various chaotic systems has been one of the main focuses of prior works, aiming to improve security in communication. Furthermore, few researchers have concentrated on the hardware implementation of these systems in order to create reliable and rapid hardware for sending and receiving data securely [23]. The summary of various works done on this topic is given below.

Çiçek et al. [24] conducted the design and implementation of an analog circuit of a secure telecommunications system based on slipping mode control (SMC). The chaos system is the jerk, which is less complex than other chaotic systems. The most significant novelty of this work is applying SMC synchronization along with the jerk chaos system, which was introduced for the first time. The results of Op-Amp-based analog circuit simulation in SMC and jerk-based synchronization demonstrated the effectiveness of the proposed method.

A method based on a four-dimensional chaotic system has been proposed by Ayub Khan et al. [25]. The chaotic system proposed is of the fractional-order type, contributing greatly to the confidentiality of information. The results obtained justified the theoretical scheme and simulation results.

Yu et al. [26] recommended a system based on hyper-chaotic theory for secure telecommunications. The proposed chaotic system is multistable four-wing memristive (FWMHS), which is five-dimensional and was used to conduct the experiments. The disturbance included in the inputs of the proposed method enhanced the security factor in telecommunications. The sliding mode control is also used in this method, the parameters of which are unknown.

A novel model for synchronization in secure telecommunications based on the fractional-order complex chaotic system has been proposed in [27]. The fractional difference function synchronization (FDFS) method used in this work yielded good results.

In [28], a new synchronization approach for utilization in internet of things (IoT) applications has been used. The design of the synchronization system in their work is based on the Lyapunov stability theorem. Synchronization is based on a nonlinear adaptive controller. In their work, the input signals are first decomposed into small segments and then combined and transmitted with chaotic signals. Subsequently, important information is separated from the chaotic signals at the receiver. The simulation results proved the effectiveness of the proposed scheme in sending and receiving confidential information.

Another work by [29] proposed the design of an Op-Amp-based analog circuit to obtain secure communication. The proposed analog circuit of the seven-dimensional chaotic system is designed and implemented. The results of the circuit simulation confirm the greater capability of the circuit designed for secure communication.

Chen et al. [30] proposed a novel methodology for the synchronization of secure telecommunications. This new technique is based on the polynomials fuzzy model applied to the Chen chaotic system. The approach proposed in this paper is implemented on several various examples, and the results showed that the receiver worked successfully in retrieving the signals transmitted by the transmitter.

Wangli He et al. [31] introduced a new approach based on quantized synchronization of neural networks. In their work, the implementation of the master and slave section of the chaos system is done on the Chua circuit. In order to perform experiments, different images for synchronization with the proposed procedure are employed, and successful results were achieved.

The idea of utilizing adaptive control for synchronization is proposed in [32]. The master and slave chaos systems discussed in this work are of the memristor type. Additionally, to enhance the security of information, an unknown parameter in the slave system is applied. The most significant contribution of this scheme is that it is simple to implement, and it helps to achieve valuable results in secure communication applications.

Ouannas et al. [33] adopted linear and nonlinear controllers to synchronize in secure telecommunications applications. The improved Robinovich chaotic system employed in this study is of the fractional order type. The stability investigation of the proposed technique is proven by means of the Lyapunov theorem. Numerical results illustrated the effectiveness of their proposed scheme in maintaining the confidentiality of information.

In a study, Wang et al. [34] proposed a novel idea of synchronization in secure telecommunications based on neural networks. Associative memory neural networks are widely employed in various applications for synchronization. This network has been chosen based on memristor. In the Lyapunov stability theorem, two controllers with different activation functions are applied. The proposed approach ensured synchronization of drive and response systems in a finite time.

Jing Wang et al. [35] conducted the implementation of analog hardware for a synchronization method; the chaotic system tested in this method is six-dimensional. In order to implement the hardware, all conditions are considered so that the theory of the proposed scheme is consistent with the simulation results.

Zirkohi et al. [36] used terminal slipping model control (TSM) for synchronization. The Duffing–Holmes oscillator is considered the first chaotic system, and the chaotic gyro oscillator as the second chaotic system. Uncertainty, unknown parameters, and ultimately external disturbances are taken into account in both chaotic systems. In the controller section, Chebyshev polynomials are applied to approximate the master and slave systems.

In this paper, a robust adaptive controller was proposed for multi-mode synchronization of chaotic systems. In the proposed method, both slave and master systems have uncertainty, disturbance, unknown parameters, and time-varying delay characteristics. By defining a suitable Lyapunov function, rules for updating parameters, time delays, and estimation errors of uncertainty and disturbance bounds were determined. The proposed controller guarantees that convergence of disturbance and uncertainty bounds estimation error and synchronization error to zero. To prevent the chattering phenomenon, the control law is a continuous function. By using the masking method and using chaotic signals as a carrier signal, the security of communication channels was improved. In the provided example, a 3D Chua system was chosen as the master system, and two Rössler systems were chosen as the slave system. Given the proper and quick reconstruction of message signals and also the convergence of all errors to zero, it was shown that the proposed method has the ability to obtain better performance for synchronization of chaotic systems.

In time-delayed chaotic systems, little work has been done on delay uncertainty with parametric uncertainties, external perturbations, and uncertainty in modeling the multi-state synchronization problems of chaotic systems. The novelties of this paper are as follows:

(1) synchronization of chaotic systems with unknown time delays;
(2) synchronization of chaotic systems in the presence of disturbance and uncertainty with unknown boundaries and variable parameters;
(3) guarantee of convergence of tracking errors and parameters estimation to zero;
(4) Determining the rules for updating parameters, time delays, and disturbance and uncertainty boundaries.

The paper is organized as follows. First, the concept of multi-state synchronization in the presence of perturbation and uncertainty is described. Then, the essential theorems to prove the convergence of errors to zero are explained. Adaptive rules for updating parameters and delays are achieved. Finally, the concept of masking the synchronization of three chaotic systems and its application in secure communication is presented.

## 2. Multi-State Synchronization of Chaotic Systems in the Presence of Disturbance and Uncertainty

**Figure 1:**
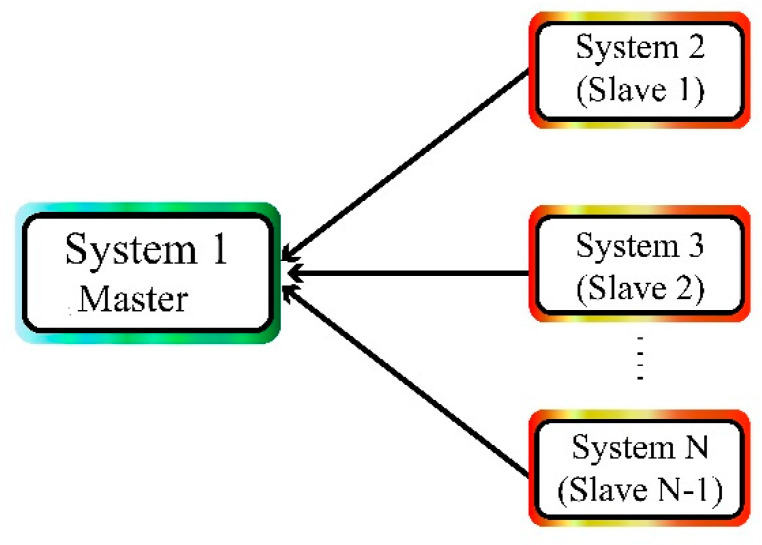
Synchronization of the master system with multiple slave systems.

In multi-state synchronization of chaotic systems, a master chaotic system is synchronized with multiple chaotic systems. Figure 1 shows the synchronization of the master system with multiple system slave systems.

We represent the driver system with the following equation [30]:

(1) $${\dot{x}}_{1}\left(t\right)\;=f_{1}\left(x_{1}\right)+F_{1}\left(x_{1}\left(t-\tau_{1}\right)\right)+H_{1}\left(x_{1}\right)\theta_{1}\left(t\right)+\Delta f_{1}\left(x_{1}\right)+D_{1}\left(t\right)$$

The N-1 slave systems control function can be represented as [30]

(2) $${\dot{x}}_{i}\left(t\right)=f_{i}\left(x_{i}\right)+F_{i}\left(x_{i}\left(t-\tau_{i}\right)\right)+H_{i}\left(x_{i}\right)\theta_{i}\left(t\right)+\Delta f_{i}\left(x_{i}\right)+D_{i}\left(t\right).\;\;i=2,3,\ldots ,N$$

where $x_{i}\left(t\right)\;={\left[x_{i1},x_{i2},\ldots ,x_{in}\right]}^{T}$ is the state vector of i-th system, $f_{i}(x_{i}\left(t\right))\;={\left[f_{i1},f_{i2},\ldots ,f_{in}\right]}^{T}$ is a continuous function,$\;F_{i}\left(x_{i}\left(t-\tau_{i}\right)\right)$ is a continuous function with Lipschitz [30] condition and constant $h_{i},$ $\tau_{i}$ is the unknown variable system delay, and $H_{i}(x_{i}\left(t\right))\;={\left[H_{i1},H_{i2},\ldots ,H_{in}\right]}^{T}$ is a matrix function. Moreover, $\theta_{i}\left(t\right)\;={\left[\theta_{i1},\theta_{i2},\ldots ,\theta_{in}\right]}^{T}$ are the main parameters with unknown step changes, $\Delta f_{i}\left(x_{i}\right)$ uncertainties, and bounded disturbance $D_{i}\left(t\right)$.

Based on Equations (1) and (2), the synchronization of chaotic system with control function is as follows:

(3) $$\left\{ \begin{array}{c} {\dot{x}}_{1}=f_{1}\left(x_{1}\right)+F_{1}\left(x_{1}\left(t-\tau_{1}\right)\right)+H_{1}\left(x_{1}\right)\theta_{1}\left(t\right)+\Delta f_{1}\left(x_{1}\right)+D_{1}\left(t\right).\\ {\dot{x}}_{2}=f_{2}\left(x_{2}\right)+F_{2}\left(x_{2}\left(t-\tau_{2}\right)\right)+H_{2}\left(x_{2}\right)\theta_{2}\left(t\right)+\Delta f_{2}\left(x_{2}\right)+D_{2}\left(t\right)+u_{1}\left(t\right).\\ \vdots \\ {\dot{x}}_{N}=f_{N}\left(x_{N}\right)+F_{N}\left(x_{N}\left(t-\tau_{N}\right)\right)+H_{N}\left(x_{N}\right)\theta_{N}\left(t\right)+\Delta f_{N}\left(x_{N}\right)+D_{N}\left(t\right)+u_{N-1}\left(t\right). \end{array}\right.$$

in which it is assumed that $u_{i-1}\left(t\right)={\left[u_{i-1.1}\left(t\right),u_{i-1.2}\left(t\right),\ldots ,u_{i-1.n}\left(t\right)\right]}^{T}$ is the *i*-th slave system control function, uncertainties and disturbance have unknown bounds,

(4) $$\left|\Delta f_{i}\left(x_{i}\right)\right|\leq \gamma_{i}g_{i}(x_{i})\;,\;\left|D_{i}\left(t\right)\right|\;\leq d_{i}\;i\;=\;1,2,\ldots ,N.$$

In Equation (4) $\gamma_{i}$ and $d_{i}$ are unknown constants, $g_{i}(x_{i})$ is a known function, and in few cases $g_{i}(x_{i})\;=\;\left|x_{i}\right|$.

In multi-state synchronization, the synchronization error is defined as [37]

(5) $$e_{i-1}\left(t\right)=x_{i}\left(t\right)-x_{1}\left(t\right).\;i=2,3,\ldots N.$$

Hence, the error dynamics can be represented as [37]

(6) $$\begin{array}{ll} {\dot{e}}_{i-1}\left(t\right)= & f_{i}\left(x_{i}\right)+F_{i}\left(x_{i}\left(t-\tau_{i}\right)\right)-f_{1}\left(x_{1}\right)\\ & -F_{1}\left(x_{1}\left(t-\tau_{1}\right)\right)+H_{i}\left(x_{i}\right)\theta_{i}\left(t\right)\\ & -H_{1}\left(x_{1}\right)\theta_{1}\left(t\right)+\;\Delta f_{i}\left(x_{i}\right)\\ & -\Delta f_{1}\left(x_{1}\right)+\;D_{i}\left(t\right)-D_{1}\left(t\right)\\ & +u_{i-1}\left(t\right).\;i\;=\;2,3,\;\ldots ,N-1. \end{array}$$

Assuming that the control function is defined as

(7) $$\begin{array}{ll} u_{i-1}(t)\;=\; & -f_{i}\left(x_{i}\right)+f_{1}\left(x_{1}\right)-H_{i}\left(x_{i}\right){\hat{\theta }}_{i}\left(t\right)\\ & +H_{1}\left(x_{1}\right){\hat{\theta }}_{1}\left(t\right)+K_{i-1}e_{i-1}\\ & -F_{i}\left(x_{i}\left(t-{\hat{\tau }}_{i}\right)\right)\\ & +F_{1}\left(x_{1}\left(t-{\hat{\tau }}_{1}\right)\right)+{\bar{u}}_{i-1}\left(t\right).\;\\ & i\;=\;2,3,\;\ldots ,N-1, \end{array}$$

The control function is selected in such a way that first it removes well-determined sentences ($f_{1}\left(x_{1}\right)\;,f_{i}\left(x_{i}\right)$) from the error dynamics, and then it approximates the sentences with variable parameters ($H_{1}\left(x_{1}\right){\hat{\theta }}_{1}\left(t\right)$ and $F_{i}\left(x_{i}\left(t-{\hat{\tau }}_{i}\right)\right)$). To stabilize the system, it exploits the $K_{i-1}e_{i-1}$ state feedback, and finally the sentence ${\bar{u}}_{i-1}\left(t\right)$ determines the proper estimation of the disturbance and uncertainty boundaries.

Where ${\hat{\theta }}_{i}\left(t\right)$ and ${\hat{\tau }}_{i}\left(t\right)$ are the estimates of $\theta_{i}\left(t\right)$ and $\tau_{i}\left(t\right)$, and ${\bar{u}}_{i-1}\left(t\right)$ is a part of the control function (introduced later in the paper), the feedback gain matrix is defined as

(8) $$K_{i-1}=-\mathrm{diag}\left(k_{i-1,1},k_{i-1,2},\ldots ,k_{i-1,n}\right)\cdot \;k_{i-1,j}>0\;j=1,2,\ldots ,n.$$

$K_{i-1}$ is a diagonal matrix with negative elements that result in the synchronization error to converge to zero in the design.

Plugging the control function into Equation (6), the errors dynamics read as

(9) $$\begin{array}{c} {\overset{\text{˙}}{e}}_{i-1}\left(t\right)\;=H_{i}\left(x_{i}\right){\tilde{\theta }}_{i}\left(t\right)-H_{1}\left(x_{1}\right){\tilde{\theta }}_{1}\left(t\right)+\\ \Delta f_{i}\left(x_{i}\right)-\Delta f_{1}\left(x_{1}\right)+F_{i}\left(x_{i}\left(t-\tau_{i}\right)\right)-F_{i}\left(x_{i}\left(t-{\hat{\tau }}_{i}\right)\right)-\\ F_{1}\left(x_{1}\left(t-\tau_{1}\right)\right)+F_{1}\left(x_{1}\left(t-{\hat{\tau }}_{1}\right)\right)+D_{i}\left(t\right)-D_{1}\left(t\right)+\\ K_{i-1}e_{i-1}+{\bar{u}}_{i-1}\left(t\right).i=2,3,\;\cdots ,N-1 \end{array}$$

where ${\tilde{\theta }}_{i}\left(t\right)\;=\theta_{i}\left(t\right)-{\hat{\theta }}_{i}\left(t\right)$ is the approximation error.

**Theorem** **1.** *If the derivative of function*f(t)*is bounded in*(a,b)*i.e.,*$\left|\frac{df}{dt}\right|\leq M$*, then*f(t)*is Lipschitz.*

**Proof** **of** **Theorem** **1.** Considering the mean value theorem,

(10) $$\forall t_{1},t_{2}\in \left(a,b\right)\;\exists c\in \left(a,b\right):\;\frac{f\left(t_{1}\right)-f\left(t_{2}\right)}{t_{1}-t_{2}}=f^{\prime }\left(c\right),$$

$$⟹\left|f\left(t_{1}\right)-f\left(t_{2}\right)\right|=\left|t_{1}-t_{2}\right|\left|f^{\prime }\left(c\right)\right|\leq \left|t_{1}-t_{2}\right|\begin{array}{c} sub\\ t\in \left(a,b\right) \end{array}\left|f^{\prime }\left(c\right)\right|\leq M\left|t_{1}-t_{2}\right|.$$

**Theorem** **2.** In a chaotic system, all the state variables are Lipschitz.

**Proof** **of** **Theorem** **2.** Considering a chaotic system characteristic, all of its state variables are bounded. On the other hand, the derivative of state variables is nothing but a set of addition, subtraction, and multiplication operations. Hence, the state variable derivatives are bounded so according to Theorem 1, the state variables are Lipschitz with $\delta_{i}$ constant, which in turn implies

(11) $$\forall t_{1},t_{2}\in R\;\exists \delta_{i}>0\;:\;\left|x_{i}\left(t_{1}\right)-x_{i}\left(t_{2}\right)\right|\leq \delta_{i}\left|t_{1}-t_{2}\right|.$$

where $x_{i}\left(t\right)$ is the state vector of *i-*th system.

**Theorem** **3.** The errors dynamics System (6), controlled by (46), using updating Rules (29)–(34) is stable. Moreover, the synchronization errors assuming uncertainty and disturbance will converge to zero.

**Proof** **of** **Theorem** **3.** Defining the Lyapunov function as

(12) $$V\;=\;\frac{1}{2}(V_{e}+\;V_{\theta }+V_{\gamma }+V_{d}+V_{\tau }).$$

where

(13) $$V_{e}\;=\;\sum_{i\;=\;2}^{N}e_{i-1}^{T}e_{i-1}\;,\;V_{\theta }\;=\sum_{i\;=\;1}^{N}{\tilde{\theta }}_{i}^{T}{\tilde{\theta }}_{i}.$$

(14) $$V_{\gamma }\;=\;\sum_{i\;=\;2}^{N}{\tilde{\gamma }}_{i}{}^{2}+{\tilde{\gamma }}_{1}{}^{2},\;V_{d}\;=\;\sum_{i\;=\;1}^{N}{\tilde{d}}_{i}{}^{2},\;V_{\tau }\;=\;\sum_{i\;=\;1}^{N}\vartheta_{i}{\tilde{\tau }}_{i}{}^{2}\;\vartheta_{i}>0.$$

In Equation (14), ${\tilde{\gamma }}_{i}=\gamma_{i}-{\hat{\gamma }}_{i},{\tilde{d}}_{i}\;={\;d}_{i}-{\hat{d}}_{i},\;{\tilde{\tau }}_{i}\;=\;\tau_{i}-{\hat{\tau }}_{i}$ are the estimation errors. Computing the Lyapunov function derivative,

(15) $$\begin{array}{ll} \dot{V}=\sum_{i=2}^{N}[e_{i-1}^{T} & (H_{i}\left(x_{i}\right){\tilde{\theta }}_{i}-H_{1}\left(x_{1}\right){\tilde{\theta }}_{1}+\Delta f_{i}\left(x_{i}\right)\\ & -\Delta f_{1}\left(x_{1}\right)+D_{i}\left(t\right)-D_{1}\left(t\right)\\ & +\frac{1}{2}\left(K_{i-1}+K_{i-1}{}^{T}\right)e_{i-1}\\ & +F_{i}\left(x_{i}\left(t-\tau_{i}\right)\right)\\ & -F_{i}\left(x_{i}\left(t-{\hat{\tau }}_{i}\right)\right)\\ & -F_{1}\left(x_{1}\left(t-\tau_{1}\right)\right)\\ & +F_{1}\left(x_{1}\left(t-{\hat{\tau }}_{1}\right)\right)+{\bar{u}}_{i-1}\left(t\right))\\ & +{\tilde{\theta }}_{i}^{T}{\dot{\tilde{\theta }}}_{i}+{\tilde{\gamma }}_{i}{\dot{\tilde{\gamma }}}_{i}+{\tilde{d}}_{i}{\dot{\tilde{d}}}_{i}+\vartheta_{i}{\tilde{\tau }}_{i}{\dot{\tilde{\tau }}}_{i}]\\ & +{\tilde{\theta }}_{1}^{T}{\dot{\tilde{\theta }}}_{1}+{\tilde{\gamma }}_{1}{\dot{\tilde{\gamma }}}_{1}+{\tilde{d}}_{1}{\dot{\tilde{d}}}_{1}\\ & +\vartheta_{1}{\tilde{\tau }}_{1}{\dot{\tilde{\tau }}}_{1} \end{array}$$

The adapting rules of system parameters are determined as

(16) $$\begin{array}{ll} {\dot{\tilde{\theta }}}_{i}\;=\;-(H_{i}{\left(x_{i}\right)}^{T} & e_{i-1}+\sigma_{i}{\tilde{\theta }}_{i}\;).\;\sigma_{i}>0\;\\ & i\;=\;2,3,\ldots ,N \end{array}$$

(17) $${\dot{\tilde{\theta }}}_{1}\;=\;\sum_{i\;=\;2}^{N-1}H_{1}{\left(x_{1}\right)}^{T}e_{i-1}-\sigma_{1}{\tilde{\theta }}_{1}.\;\sigma_{1}>0.$$

If $\theta_{i}s$ are constant, then their derivatives are zero (${\dot{\theta }}_{i}\;=\;0)$, and the update rules for the parameters estimation are computed as

(18) $$\begin{array}{ll} {\dot{\hat{\theta }}}_{i}\;=\;H_{i}{\left(x_{i}\right)}^{T}e_{i-1}+\sigma_{i}{\tilde{\theta }}_{i}.\;\sigma_{i}>0\\ & i\;=2,3,\ldots ,N \end{array}$$

(19) $${\dot{\hat{\theta }}}_{1}=-\sum_{i=2}^{N-1}H_{1}{\left(x_{1}\right)}^{T}e_{i-1}+\sigma_{1}{\tilde{\theta }}_{1}.\;\sigma_{1}>0$$

Plugging the updating rules (16–17) in Equation (15) yields

(20) $$\begin{array}{ll} \dot{V}=\sum_{i=2}^{N}[e_{i-1}^{T} & (\Delta f_{i}(x_{i})-\Delta f_{1}(x_{1})+D_{i}(t)-D_{1}(t)+K_{i-1}e_{i-1}\\ & +F_{i}\left(x_{i}\left(t-\tau_{i}\right)\right)-F_{i}\left(x_{i}\left(t-{\hat{\tau }}_{i}\right)\right)-F_{1}\left(x_{1}\left(t-\tau_{1}\right)\right)\\ & +F_{1}\left(x_{1}\left(t-{\hat{\tau }}_{1}\right)\right)+{\bar{u}}_{i-1}\left(t\right))+{\tilde{\gamma }}_{i}{\dot{\tilde{\gamma }}}_{i}+{\tilde{d}}_{i}{\dot{\tilde{d}}}_{i}+\vartheta_{i}{\tilde{\tau }}_{i}{\dot{\tilde{\tau }}}_{i}]+{\tilde{\gamma }}_{1}{\dot{\tilde{\gamma }}}_{1}\\ & +{\tilde{d}}_{1}{\dot{\tilde{d}}}_{1}+\vartheta_{1}{\tilde{\tau }}_{1}{\dot{\tilde{\tau }}}_{1}-\sum_{i\;=1}^{N}\sigma_{i}{\tilde{\theta }}_{i}^{T}{\tilde{\theta }}_{i} \end{array}$$

If $\Delta f_{i}{}^{j},F_{i}{}^{j},\;D_{i}{}^{j},\;e_{i-1}^{j},$ and ${\bar{u}}_{i-1}{}^{j}$ are the *j*-th component of vectors $\Delta f_{i},F_{i},\;D_{i},e_{i-1}$, and ${\bar{u}}_{i-1}\left(t\right)$, respectively, then

(21) $$\begin{array}{ll} \dot{V}=\sum_{i=2}^{N}\sum_{j=1}^{n}e_{i-1}^{j} & (\Delta f_{i}{}^{j}-\Delta f_{1}{}^{j}+D_{i}{}^{j}-D_{1}{}^{j}\\ & +F_{i}^{j}\left(x_{i}\left(t-\tau_{i}\right)\right)\\ & -F_{i}^{j}\left(x_{i}\left(t-{\hat{\tau }}_{i}\right)\right)\\ & -F_{1}^{j}\left(x_{1}\left(t-\tau_{1}\right)\right)\\ & +F_{1}^{j}\left(x_{1}\left(t-{\hat{\tau }}_{1}\right)\right)+{\bar{u}}_{i-1}{}^{j}\\ & +\sum_{i\;=\;2}^{N}\left({\tilde{\gamma }}_{i}{\dot{\tilde{\gamma }}}_{i}+{\tilde{d}}_{i}{\dot{\tilde{d}}}_{i}+\vartheta_{i}{\tilde{\tau }}_{i}{\dot{\tilde{\tau }}}_{i}\right)\\ & +\sum_{i\;=\;2}^{N}\frac{1}{2}e_{i-1}^{T}K_{i-1}e_{i-1}+{\tilde{\gamma }}_{1}{\dot{\tilde{\gamma }}}_{1}\\ & +{\tilde{d}}_{1}{\dot{\tilde{d}}}_{1}+\vartheta_{1}{\tilde{\tau }}_{1}{\dot{\tilde{\tau }}}_{1}\\ & -\sum_{i\;=\;1}^{N}\sigma_{i}{\tilde{\theta }}_{i}^{T}{\tilde{\theta }}_{i}. \end{array}$$

Therefore,

(22) $$\begin{array}{ll} \dot{V}\leq \sum_{i\;=\;2}^{N}\sum_{j\;=\;1}^{n}[\left|e_{i-1}^{j}\right| & (\left|\Delta f_{i}{}^{j}\right|+\left|\Delta f_{1}{}^{j}\right|+\left|D_{i}{}^{j}\right|\\ & +\left|D_{1}{}^{j}\right|\\ & +|F_{i}^{j}\left(x_{i}\left(t-\tau_{i}\right)\right)\\ & -F_{i}^{j}\left(x_{i}\left(t-{\hat{\tau }}_{i}\right)\right)|\\ & +|F_{1}^{j}\left(x_{1}\left(t-{\hat{\tau }}_{1}\right)\right)\\ & -F_{1}^{j}\left(x_{1}\left(t-\tau_{1}\right)\right)|)\\ & +e_{i-1}^{j}{\bar{u}}_{i-1}{}^{j}]\\ & +\sum_{i\;=\;2}^{N}\left({\tilde{\gamma }}_{i}{\dot{\tilde{\gamma }}}_{i}+{\tilde{d}}_{i}{\dot{\tilde{d}}}_{i}+\vartheta_{i}{\tilde{\tau }}_{i}{\dot{\tilde{\tau }}}_{i}\right)\\ & +\sum_{i\;=\;2}^{N}e_{i-1}^{T}K_{i-1}e_{i-1}+{\tilde{\gamma }}_{1}{\dot{\tilde{\gamma }}}_{1}\\ & +{\tilde{d}}_{1}{\dot{\tilde{d}}}_{1}+\vartheta_{1}{\tilde{\tau }}_{1}{\dot{\tilde{\tau }}}_{1}\\ & -\sum_{i\;=\;1}^{N}\sigma_{i}{\tilde{\theta }}_{i}^{T}{\tilde{\theta }}_{i}. \end{array}$$

In Equation (22), bounds of disturbance and uncertainty can be applied on components $\Delta f_{i}$ and $D_{i}\left(t\right)$ as follows:

(23) $$\left|\Delta f_{i}{}^{j}\right|\leq \underset{j}{max}\left|\Delta f_{i}{}^{j}\right|\leq \left|\Delta f_{i}\left(x_{i}\right)\right|\leq \gamma_{i}g_{i}(x_{i}).$$

(24) $$\left|D_{i}{}^{j}\left(t\right)\right|\leq \underset{j}{max}\left|D_{i}{}^{j}\left(t\right)\right|\leq \left|D_{i}\left(t\right)\right|\leq d_{i}.$$

Since $F_{i}\left(x_{i}\left(t-\tau_{i}\right)\right)$ and $x_{i}\left(t-\tau_{i}\right)$ are Lipschitz, their components are Lipschitz as well:

(25) $$\begin{array}{c} \left|F_{i}^{j}\left(x_{i}\left(t-\tau_{i}\right)\right)-F_{i}^{j}\left(x_{i}\left(t-{\hat{\tau }}_{i}\right)\right)\right|\leq h_{i}\left|x_{i}\left(t-\tau_{i}\right)-x_{i}\left(t-{\hat{\tau }}_{i}\right)\right|\\ \leq h_{i}\delta_{i}\left|\left(t-\tau_{i}\right)-\left(t-{\hat{\tau }}_{i}\right)\right|=h_{i}\delta_{i}\left|\tau_{i}-{\hat{\tau }}_{i}\right|\;=\;h_{i}\delta_{i}\left|{\tilde{\tau }}_{i}\right|\\ \;=\;\vartheta_{i}\left|{\tilde{\tau }}_{i}\right| \end{array}$$

$$\vartheta_{i}≜h_{i}\delta_{i}.$$

Substituting the above equation in Equation (22) yields

(26) $$\begin{array}{ll} \dot{V}\leq \sum_{i\;=\;2}^{N}\sum_{j\;=\;1}^{n}[\left|e_{i-1}^{j}\right| & (\gamma_{i}g_{i}(x_{i})+\gamma_{1}g_{1}(x_{1})+d_{i}\\ & +d_{1})+\vartheta_{i}\left|{\tilde{\tau }}_{i}\right|+\vartheta_{1}\left|{\tilde{\tau }}_{1}\right|)\\ & +e_{i-1}^{j}{\bar{u}}_{i-1}{}^{j}]\\ & +\sum_{i\;=\;2}^{N}\left({\tilde{\gamma }}_{i}{\dot{\tilde{\gamma }}}_{i}+{\tilde{d}}_{i}{\dot{\tilde{d}}}_{i}+\vartheta_{i}{\tilde{\tau }}_{i}{\dot{\tilde{\tau }}}_{i}\right)\\ & +\sum_{i\;=\;2}^{N}e_{i-1}^{T}K_{i-1}e_{i-1}+{\tilde{\gamma }}_{1}{\dot{\tilde{\gamma }}}_{1}\\ & +{\tilde{d}}_{1}{\dot{\tilde{d}}}_{1}+\vartheta_{1}{\tilde{\tau }}_{1}{\dot{\tilde{\tau }}}_{1}-\sum_{i\;=\;1}^{N}\sigma_{i}{\tilde{\theta }}_{i}^{T}{\tilde{\theta }}_{i}. \end{array}$$

Defining ${\bar{u}}_{i-1}{}^{j}\left(t\right)$ as

(27) $${\bar{u}}_{i-1}{}^{j}\left(t\right)\;=\;-\left({\hat{\gamma }}_{i}g_{i}(x_{i}\right)+{\hat{\gamma }}_{1}g_{1}(x_{1})+{\hat{d}}_{i}+{\hat{d}}_{1})\cdot \mathrm{sgn}\left(e_{i-1}^{j}\right).$$

yields

(28) $$\begin{array}{ll} \dot{V}\leq \sum_{i\;=\;2}^{N}\sum_{j\;=\;1}^{n}[\left|e_{i-1}^{j}\right| & ({\tilde{\gamma }}_{i}g_{i}(x_{i})+{\tilde{\gamma }}_{1}g_{1}(x_{1})+{\tilde{d}}_{i}\\ & +{\tilde{d}}_{1}+\vartheta_{i}\left|{\tilde{\tau }}_{i}\right|+\vartheta_{1}\left|{\tilde{\tau }}_{1}\right|)]\\ & +\sum_{i\;=\;1}^{N}({\tilde{\gamma }}_{i}{\dot{\tilde{\gamma }}}_{i}+{\tilde{d}}_{i}{\dot{\tilde{d}}}_{i}+\vartheta_{i}{\tilde{\tau }}_{i}{\dot{\tilde{\tau }}}_{i}\\ & -\sigma_{i}{\tilde{\theta }}_{i}^{T}{\tilde{\theta }}_{i})+\sum_{i\;=\;2}^{N}e_{i-1}^{T}K_{i-1}e_{i-1}\\ & =\;\sum_{j\;=\;1}^{n}\left(\left|e_{i-1}^{j}\right|{\tilde{\gamma }}_{i}g_{i}(x_{i}\right)+{\tilde{\gamma }}_{i}{\dot{\tilde{\gamma }}}_{i})\\ & +\sum_{j\;=\;1}^{n}(\left|e_{i-1}^{j}\right|\vartheta_{i}\left|{\tilde{\tau }}_{i}\right|+\vartheta_{i}{\tilde{\tau }}_{i}{\dot{\tilde{\tau }}}_{i})\\ & +\sum_{j\;=\;1}^{n}\left(\left|e_{i-1}^{j}\right|{\tilde{d}}_{i}+{\tilde{d}}_{i}{\dot{\tilde{d}}}_{i}\right)+({\tilde{\gamma }}_{1}{\dot{\tilde{\gamma }}}_{1}\\ & +\sum_{i\;=\;1}^{N}\sum_{j\;=\;1}^{n}\left|e_{i-1}^{j}\right|{\tilde{\gamma }}_{1}g_{1}(x_{1}))\\ & +({\tilde{d}}_{1}{\dot{\tilde{d}}}_{1}+\sum_{i\;=\;1}^{N}\sum_{j\;=\;1}^{n}\left|e_{i-1}^{j}\right|{\tilde{d}}_{1})\\ & +(\vartheta_{1}{\tilde{\tau }}_{1}{\dot{\tilde{\tau }}}_{1}\\ & +\sum_{i\;=\;1}^{N}\sum_{j\;=\;1}^{n}\left|e_{i-1}^{j}\right|\vartheta_{1}\left|{\tilde{\tau }}_{1}\right|)\\ & -\sum_{i\;=\;1}^{N}\sigma_{i}{\tilde{\theta }}_{i}^{T}{\tilde{\theta }}_{i}\\ & +\sum_{i\;=\;2}^{N}e_{i-1}^{T}K_{i-1}e_{i-1}. \end{array}$$

To determine the update rules, we determine the derivatives of the signals in (28) in such a way that $\dot{V}$ is negative. To that end, the updating rules can be set as follows:

(29) $$\sum_{j\;=\;1}^{n}\left(\left|e_{i-1}^{j}\right|\vartheta_{i}\left|{\tilde{\tau }}_{i}\right|+\vartheta_{i}{\tilde{\tau }}_{i}{\dot{\tilde{\tau }}}_{i}\right)\;=\;-\omega_{i}{\tilde{\tau }}_{i}{}^{2}\Rightarrow {\dot{\tilde{\tau }}}_{i}\;=\;-\sum_{j\;=\;1}^{n}\left|e_{i-1}^{j}\right|\mathrm{sgn}\left({\tilde{\tau }}_{i}\right)-\rho_{i}{\tilde{\tau }}_{i}\cdot \rho_{i}\;\;=\;\frac{\omega_{i}}{\vartheta_{i}}.$$

(30) $$\vartheta_{1}{\tilde{\tau }}_{1}{\dot{\tilde{\tau }}}_{1}+\sum_{i\;=\;1}^{N}\sum_{j\;=\;1}^{n}\left|e_{i-1}^{j}\right|\vartheta_{1}\left|{\tilde{\tau }}_{1}\right|\;=\;-{\tilde{\tau }}_{1}{}^{2}\Rightarrow {\dot{\tilde{\tau }}}_{1}\;\;=\;-\sum_{i\;=\;1}^{N}\sum_{j\;=\;1}^{n}\left|e_{i-1}^{j}\right|\mathrm{sgn}\left({\tilde{\tau }}_{1}\right)-\rho_{1}{\tilde{\tau }}_{1}\cdot \;\rho_{1}\;=\;\frac{\omega_{1}}{\vartheta_{1}}.$$

(31) $$\sum_{j\;=\;1}^{n}\left(\left|e_{i-1}^{j}\right|{\tilde{\gamma }}_{i}g_{i}(x_{i}\right)+{\tilde{\gamma }}_{i}{\dot{\tilde{\gamma }}}_{i})\;=\;-\alpha_{i}{\tilde{\gamma }}_{i}{}^{2}\;\Rightarrow {\dot{\tilde{\gamma }}}_{i}\;\;=\;-\sum_{j\;=\;1}^{n}\left|e_{i-1}^{j}\right|g_{i}(x_{i})-\alpha_{i}{\tilde{\gamma }}_{i}.$$

(32) $${\tilde{\gamma }}_{1}{\dot{\tilde{\gamma }}}_{1}+\sum_{i\;=\;1}^{N}\sum_{j\;=\;1}^{n}\left|e_{i-1}^{j}\right|{\tilde{\gamma }}_{1}g_{1}(x_{1})\;=\;-\alpha_{1}{\tilde{\gamma }}_{1}{}^{2}\;\Rightarrow {\dot{\tilde{\gamma }}}_{1}\;\;=\;-g_{1}(x_{1})\sum_{i\;=\;1}^{N}\sum_{j\;=\;1}^{n}\left|e_{i-1}^{j}\right|\;-\alpha_{1}{\tilde{\gamma }}_{1}.\;$$

(33) $$\sum_{j\;=\;1}^{n}\left(\left|e_{i-1}^{j}\right|{\tilde{d}}_{i}+{\tilde{d}}_{i}{\dot{\tilde{d}}}_{i}\right)\;=\;-\beta_{i}{\tilde{d}}_{i}{}^{2}\Rightarrow {\dot{\tilde{d}}}_{i}\;\;=\;-\sum_{j\;=\;1}^{n}\left|e_{i-1}^{j}\right|-\beta_{i}{\tilde{d}}_{i}.$$

(34) $${\tilde{d}}_{1}{\dot{\tilde{d}}}_{1}+\sum_{i\;=\;1}^{N}\sum_{j\;=\;1}^{n}\left|e_{i-1}^{j}\right|{\tilde{d}}_{1}\;=\;-\beta_{1}{\tilde{d}}_{1}{}^{2}\Rightarrow {\dot{\tilde{d}}}_{1}\;=\;-\sum_{i\;=\;1}^{N}\sum_{j\;=\;1}^{n}\left|e_{i-1}^{j}\right|-\beta_{1}{\tilde{d}}_{1}.$$

where $\alpha_{i}$, $\beta_{i}$, and $\omega_{i}$ are positive values. Substituting the update rules (Equations (29)–(34)) in Equation (28) yields

(35) $$\dot{V}\leq \sum_{i\;=\;2}^{N}e_{i-1}^{T}K_{i-1}e_{i-1}-\sum_{i\;=\;1}^{N}(\alpha_{i}{\tilde{\gamma }}_{i}{}^{2}+\beta_{i}{\tilde{d}}_{i}{}^{2}+\omega_{i}{\tilde{\tau }}_{i}{}^{2}+\sigma_{i}{\tilde{\theta }}_{i}^{T}{\tilde{\theta }}_{i}).$$

Given that the matrixes $K_{i-1}$ are diagonal and have negative elements (Hurwitz). Therefore:$\;e_{i-1}^{T}K_{i-1}e_{i-1}<0$.

Defined *μ* as

(36) $$\mu \;=\;\underset{i.j}{min}\left(\alpha_{i},\beta_{i},\;\sigma_{i}\;,\omega_{i},k_{i-1,j}\right)>0$$

Using (35) for the derivative of the Lyapunov function, the following inequality is established:

(37) $$\dot{V}\leq -\mu V\Rightarrow V\left(t\right)\leq V\left(0\right)e^{-\mu t}\Rightarrow V\left(t\right)\to 0$$

As the values of the parameters $\alpha_{i},\beta_{i},\;\sigma_{i}\;,\omega_{i},k_{i-1,j}$ augment, the convergence speed of synchronization errors and estimation error signals to zero increases.

Therefore, the system stability is proved. Additionally, the convergence of synchronization errors to zero in the presence of time delay, uncertainty, and disturbance is guaranteed. The update rules for delays estimation, disturbance bounds, and uncertainty are as follows

(38) $$\dot{{\hat{\tau }}_{i}}\;=\;\sum_{j\;=\;1}^{n}\left|e_{i-1}^{j}\right|\mathrm{sgn}\left({\tilde{\tau }}_{i}\right)+\rho_{i}{\tilde{\tau }}_{i}.i\;=\;2,3,\ldots ,N$$

(39) $$\dot{{\hat{\tau }}_{1}}=\;\sum_{i\;=\;1}^{N}\sum_{j\;=\;1}^{n}\left|e_{i-1}^{j}\right|\mathrm{sgn}\left({\tilde{\tau }}_{1}\right)+\rho_{1}{\tilde{\tau }}_{1}.$$

(40) $$\dot{{\hat{\gamma }}_{i}}\;=\;g_{i}(x_{i})\sum_{j\;=\;1}^{n}\left|e_{i-1}^{j}\right|+\alpha_{i}{\tilde{\gamma }}_{i}.i\;=\;2,3,\ldots ,N$$

(41) $$\dot{{\hat{\gamma }}_{i}}\;=\;g_{1}(x_{1})\sum_{i\;=\;1}^{N}\sum_{j\;=\;1}^{n}\left|e_{i-1}^{j}\right|+\alpha_{1}{\tilde{\gamma }}_{1}.$$

(42) $$\dot{{\hat{d}}_{i}}\;=\;\sum_{j\;=\;1}^{n}\left|e_{i-1}^{j}\right|+\beta_{i}{\tilde{d}}_{i}.\;i\;=\;2,3,\ldots ,N$$

(43) $$\dot{{\hat{d}}_{1}}\;=\sum_{i\;=\;1}^{N}\sum_{j\;=\;1}^{n}\left|e_{i-1}^{j}\right|+\beta_{1}{\tilde{d}}_{1}.$$

**Theorem** **4.** *If*$\tau_{i}\left(t\right)$*are constant values, then*${\tilde{\tau }}_{i}\left(t\right)\to 0$. In other words, the delays are identified accurately.

**Proof** **of** **Theorem** **4.** Based on Equations (29) and (30), ${\tilde{\tau }}_{i}{\dot{\tilde{\tau }}}_{i}<-\omega_{i}{\tilde{\tau }}_{i}{}^{2}\;i=1,2,3,\ldots ,N$, as t approaches infinity (t→∞),${\tilde{\tau }}_{i}\left(t\right)$ approaches zero (${\tilde{\tau }}_{i}\left(t\right)\to 0$). Therefore, estimation ${\hat{\tau }}_{i}\left(t\right)$ approaches the true value.

**Theorem** **5.** If the delays change in a step-wise manner and the steps are large enough, the update Rules (17) and (19) hold.

**Proof** **of** **Theorem 4.** Similar to Theorem 4 with the exception that t→∞ condition is replaced with condition “the step changes are large enough”.

**Note 1:** If the delays vary with time and $\left|\dot{\tau_{i}}\left(t\right)\right|\leq s_{i}$ holds with $s_{i}<1$, then the update rules in (38-39) are valid with reasonable approximation.

Considering that ${\tilde{\tau }}_{i}\left(t\right)\;=\;\tau_{i}\left(t\right)-{\hat{\tau }}_{i}\left(t\right)$, ${\dot{\hat{\tau }}}_{i}\;=\;\dot{\tau_{i}}\left(t\right)-{\dot{\tilde{\tau }}}_{i}\approx -{\dot{\tilde{\tau }}}_{i}$, the update rules (38-39) are valid with reasonable approximation. Moreover, under such conditions, update Rule (29-30) is exactly valid.

**Note 2:** If the systems vary with time, i.e., $\theta_{i}=\theta_{i}\left(t\right)$, Equations (16) and (17) hold, which allow us to apply Theorem (3) based on which Lyapunov Function (12) can be used, and its derivative satisfies Condition (35) as well. Hence, the Lyapunov function approaches zero:

(44) $$V\to 0\Rightarrow V_{\theta }=\;\sum_{i=1}^{N}{\tilde{\theta }}_{i}^{T}{\tilde{\theta }}_{i}\to 0\Rightarrow \left|{\tilde{\theta }}_{i}\right|\to 0$$

Therefore, if $\theta_{i}\left(t\right)$ is a vector function with step changes and appropriate temporal distance between the changes, the update Rules (16) and (17) hold and parameter are estimated accurately. Let $\left|\dot{\theta_{i}}\left(t\right)\right|<q_{i}\;<\;1$, then update Rules (18) and (19) are valid with reasonable accuracy.

**Note 3:** To guarantee the continuity of the control function, the following equation can be used:

(45) $${\bar{u}}_{i-1}{}^{j}\left(t\right)\;=\;-\left({\hat{\gamma }}_{i}g_{i}(x_{i}\right)+{\hat{\gamma }}_{1}g_{1}(x_{1})+{\hat{d}}_{i}+{\hat{d}}_{1})\cdot \tan h\left(\lambda e_{i-1}^{j}\right)).\lambda \geq 10$$

**Note 4:** If the uncertainties are in their typical form, i.e., $\left|\Delta f_{i}\left(x_{i}\right)\right|\leq \gamma_{i}\left|x_{i}\right|\;i\;=\;1.2.\ldots .N$.., it suffices to set $g_{i}(x_{i})\;=\;\left|x_{i}\right|$ in the update equations and the control rule.

**Note 5:** The final control function is as below:

(46) $$u_{i-1}\left(t\right)\;=\;-f_{i}\left(x_{i}\right)+f_{1}\left(x_{1}\right)-H_{i}\left(x_{i}\right){\hat{\theta }}_{i}\left(t\right)+H_{1}\left(x_{1}\right){\hat{\theta }}_{1}\left(t\right)+K_{i-1}e_{i-1}\left(t\right)\;-\left({\hat{\gamma }}_{i}g_{i}(x_{i}\right)+{\hat{\gamma }}_{1}g_{1}(x_{1})+{\hat{d}}_{i}+{\hat{d}}_{1})\cdot \tan h\left(\lambda e_{i-1}\left(t\right)\right).\lambda \geq 10$$

## 3. Application in Secure Communication Based on Chaotic Masking

In chaotic masking, an information signal is added to the linear combination of base state signals. Given that Q(t) is the primary signal carried by the master system and W(t) is the transfer message given by [38,39]:

(47) $$W\left(t\right)\;=\;Q\left(t\right)+\sum_{i\;=\;1}^{n}\eta_{i}z_{i}\left(t\right).\;$$

where $z_{i}\left(t\right)$ is the *i*-th component of the master system, and W(t) is masked using the chaotic signal. This signal is transmitted via the communication channel from sender to the receiver. Using the proposed controller, the multi-state chaotic synchronization is performed in one of its states. The received signal can be recovered using the following equation [40]:

(48) $$P\left(t\right)\;=\;W\left(t\right)-\sum_{i\;=\;1}^{n}\eta_{i}y_{i}\left(t\right).$$

where $y_{i}\left(t\right)$ is the *i*-th component of the slave system. Considering the synchronization concept, the following equation is obtained [40]:

(49) $$P\left(t\right)\;=\;Q\left(t\right)+\sum_{i\;=\;1}^{n}\eta_{i}z_{i}\left(t\right)-\sum_{i\;=\;1}^{n}\eta_{i}y_{i}\left(t\right)\;=\;Q\left(t\right)+\sum_{i\;=\;1}^{n}\eta_{i}(z_{i}\left(t\right)-y_{i}\left(t\right))\;\;=\;Q\left(t\right)+\sum_{i\;=\;1}^{n}\eta_{i}e_{i}\left(t\right)\to Q\left(t\right)$$

**Figure 2:**
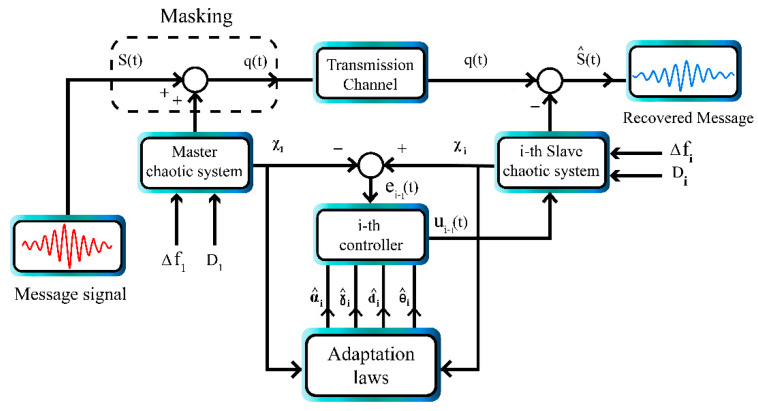
Block diagram of chaotic masking using multi-state synchronization.

Figure 2 shows the block diagram of chaotic masking using multi-state synchronization. Since we have one master and two slave systems, two independent messages are sent to the master which are encrypted, then the master is synchronized with two slaves. After the synchronization with the receiver side, the signals are decrypted and the original messages are recovered.

Using chaotic masking, we demonstrated the encryption and decryption of two sinusoidal signals using our proposed multi-state synchronization approach. The simulation is carried out with Matlab software. The encryption and decryption are applied when the chaotic signals are synchronized. To evaluate this, two sinusoidal signals are added to the master system signals. Next, the master system is synchronized with two slave systems. Finally, based on the synchronization error, the signal is decrypted and recovered at the receiver side.

## 4. Simulation and Results

We used one Chua chaotic system and two Rössler time-delayed chaotic systems as master and slaves, respectively. These systems are defined as follows:

(50) $$\left\{ \begin{array}{l} {\dot{x}}_{11}=\theta_{11}(x_{12}-x_{11}-f(x_{11})).\\ {\dot{x}}_{12}=x_{11}(t-\tau_{1})-x_{12}-x_{13}.\\ {\dot{x}}_{13}=-\theta_{12}x_{12}.\\ f(x_{11})=ax_{11}+0.5(a-b)(|x_{11}+1|)-|x_{11}-1|. \end{array}\right.$$

(51) $$\{\begin{array}{l} {\dot{x}}_{21}=-x_{22}-x_{23}+u_{11}.\\ {\dot{x}}_{22}=x_{21}\left(t-\tau_{2}\right)+\theta_{21}x_{22}+u_{12}.\;\;\;\\ {\dot{x}}_{23}=\theta_{22}x_{21}-\theta_{23}x_{23}+x_{21}x_{23}+u_{13} \end{array}$$

(52) $$\{\begin{array}{l} {\dot{x}}_{31}=-x_{32}-x_{33}+u_{21}.\\ {\dot{x}}_{32}=x_{31}\left(t-\tau_{3}\right)+\theta_{31}x_{32}+u_{22}.\;\;\;\;\\ {\dot{x}}_{33}=\theta_{32}x_{11}-\theta_{33}x_{33}+x_{31}x_{33}+u_{23}. \end{array}$$

where $\theta_{i,j},b,a$ are system parameters. The values of the parameters are set as $a\;=\;-\frac{61}{44},b\;=\;\frac{3}{4}$, $\theta_{11}\;=\;10,\theta_{12}\;=\;18,\theta_{21}\;=\;\theta_{31}\;=\;0.34,\theta_{22}\;=\;\theta_{32}\;=\;0.4,\theta_{23}\;=\;\theta_{33}\;=\;4.5\;.$

(53) $$\tau_{1}\left(t\right)=\{\begin{array}{l} 1\;0\leq t\leq 2\\ 0.3\;2<t\leq 4\\ 1\;t>4 \end{array}$$

(54) $$\tau_{2}\left(t\right)=\{\begin{array}{l} 2\;0\leq t\leq 3\\ 5\;3<t\leq 6.5\\ 3\;t>6.5 \end{array}$$

(55) $$\tau_{3}\left(t\right)=\{\begin{array}{l} 3\;0\leq t\leq 2.5\\ 5+0.4sin\left(2\pi t\right)\;2.5<t\leq 5.5\\ 10+0.6sin\left(\frac{\pi t}{2}\right)\;t>5.5 \end{array}$$

The initial values of the parameters are set as below:

(56) $${\hat{\theta }}_{1}\left(0\right)\;=\;\left[\begin{array}{c} 10\\ 10\\ \begin{array}{c} 10\\ 10 \end{array} \end{array}\right]{\hat{\theta }}_{2}\left(0\right)\;=\;\left[\begin{array}{c} 2\\ 2\\ \begin{array}{c} 2\\ 2 \end{array} \end{array}\right]{\hat{\theta }}_{3}\left(0\right)\;=\;\left[\begin{array}{c} 3\\ 3\\ \begin{array}{c} 3\\ 3 \end{array} \end{array}\right]\sigma_{i}\;=\;10\;.\;i\;=\;1.2.3.4.5$$

Under such circumstances, the parameters have step changes. In addition, the disturbance and uncertainties influencing the master and slave systems are expressed as

(57) $$\Delta f_{1}\;=\;\left[\begin{array}{c} 0.2x_{11}\sin\left(x_{11}+x_{13}\right)\\ 0.01x_{13}\sin\left(x_{11}+x_{13}\right)\\ 0.02\left(x_{11}+x_{12}\right)\cos\left(x_{11}-x_{13}\right) \end{array}\right]$$

(58) $$\Delta f_{2}=\left[\begin{array}{c} 0.5\sin\left(x_{21}-x_{22}\right)\\ 0.2x_{24}\cos\left(x_{21}-3x_{22}\right)\\ 0.5\cos\left(x_{21}+2x_{23}\right) \end{array}\right]$$

(59) $$\Delta f_{3}\;=\;\left[\begin{array}{c} 0.3x_{32}cos\left(x_{31}+x_{32}\right)\\ 0.5sin\left(x_{31}-x_{32}\right)\\ 0.4x_{32}sin\left(x_{31}+x_{33}\right) \end{array}\right]$$

(60) $$\left|\Delta f_{i}{}^{j}\right|\leq \;\underset{j}{max}\left|\Delta f_{i}{}^{j}\right|\leq \left|\Delta f_{i}\left(x_{i}\right)\right|\leq \gamma_{i}\left|x_{i}\right|$$

(61) $$D_{1}\;=\;\left[\begin{array}{c} 0.2sin\left(\frac{\pi }{3}t\right)\\ 0.1sin\left(\frac{\pi }{10}t\right)\\ 0.25sin\left(\frac{\pi }{4}t\right) \end{array}\right].D_{2}\;=\;\left[\begin{array}{c} 0.25sin\left(\frac{\pi }{2}t\right)\\ 0.15sin\left(\frac{\pi }{20}t\right)\\ 0.20sin\left(\frac{\pi }{10}t\right) \end{array}\right].D_{3}\;=\;\left[\begin{array}{c} 0.3sin\left(\frac{\pi }{30}t\right)\\ 0.2sin\left(\frac{\pi }{20}t\right)\\ 0.15sin\left(\frac{\pi }{10}t\right) \end{array}\right]$$

The controller’s parameters are chosen as

(62) $$\alpha_{i}\;=\;1,\beta_{i}\;=\;20,\;i\;=\;1,2,3\;K_{1}\;=\;K_{2}\;=\;diag\left(-20,-20,-20\right)$$

**Figure 3:**
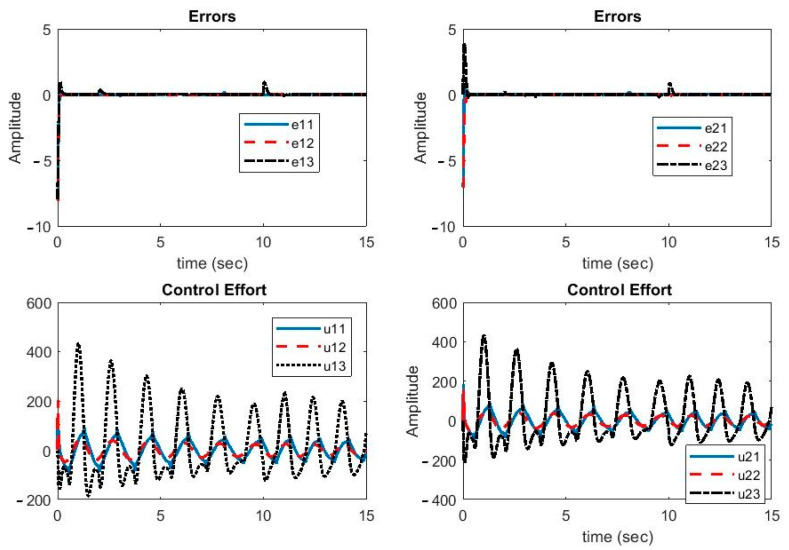
Plots of synchronization errors and control efforts.

In Figure 3, the synchronization errors and control efforts are illustrated. As can be seen, despite the parameter uncertainties and time-variable delays, the error signals were reduced to zero quickly. During large parameter changes, the error increased slightly but turned back to zero quickly. Hence, the proposed controller was robust against parameter uncertainties, external disturbance, and variable delays. Moreover, the control function was continuous and smooth, which was helpful in the implementation of the proposed method.

**Figure 4:**
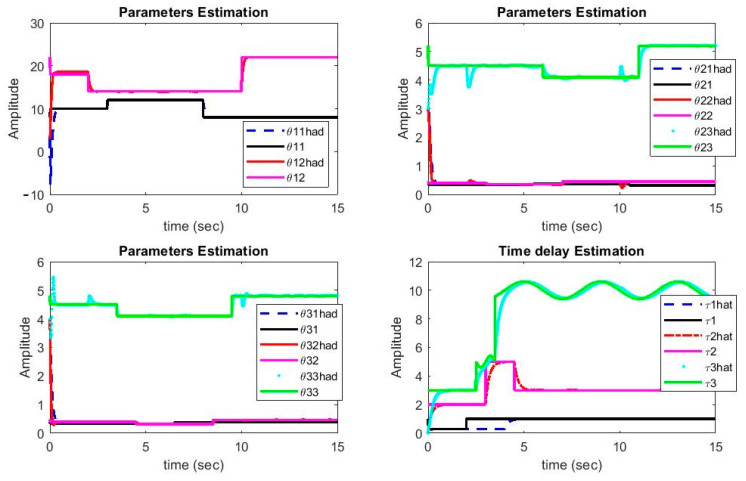
Plots of parameters and delays estimations versus time.

It can be noted from Figure 4 that the estimated parameters of Chua and Rössler systems have converged to their real values. It is evident from this figure that, despite being variable with time, the delays have also converged to their real values quickly. During the instances when delays exhibited step changes, the system parameters deviated from their real values, but they were corrected in short duration. Since Lipschitz conditions are $F_{i}\left(x_{i}\left(t-\tau_{i}\right)\right)$ functions, the parameter changes do not affect the delays significantly. On the other hand, Chua is a non-smooth system, which causes more complication in the control as well as synchronization problems.

**Figure 5:**
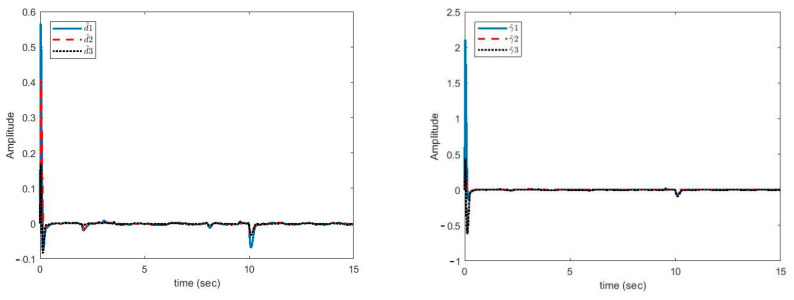
Estimation error of disturbance bounds (left). Estimation error of uncertainties bounds versus time (right).

In Figure 5, the plots of estimation error of unknown uncertainties bounds ${\tilde{\gamma }}_{i},\;i=1,2,3$ and unknown disturbances bounds ${\tilde{d}}_{i},\;i=1,2,3$ are shown.

**Figure 6:**
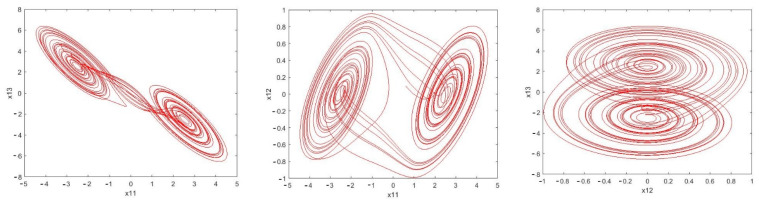
Sample phase curves of the Master system.

**Figure 7:**
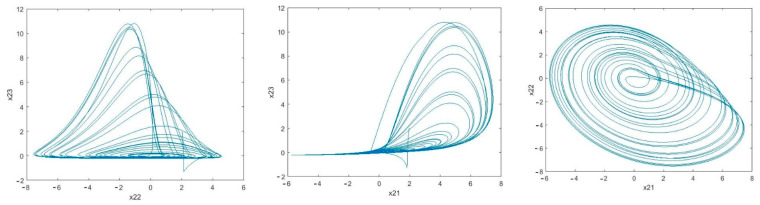
Sample phase curves of the first slave system.

**Figure 8:**
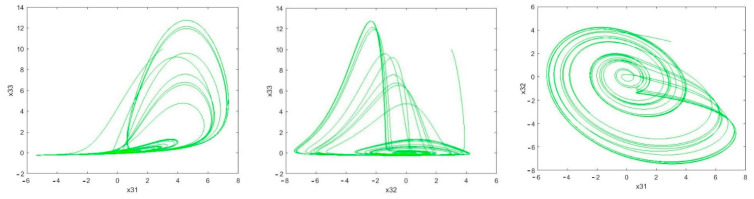
Sample phase curves of the second slave system.

It can be seen in Figure 5 that the estimation error of unknown parameter bounds and uncertainties bounds have approached zero quickly. In the tenth second, little error is observed, which is compensated in a short amount of time. Figure 6, Figure 7 and Figure 8 shows the phase curves of the slave system and indicates the chaotic behavior of the system. In spite of various changes, the behavior of the system is still chaotic.

Phase curves of the master system: Chua time-delayed chaotic system $(x_{11}-x_{13},x_{11}-x_{12},x_{12}-x_{13}$) indicating the chaotic behavior for the provided parameters.

The phase curve of the first slave system: Rössler time-delayed chaotic systems ($x_{21}-xx_{22}-x_{23},x_{21}-x_{23}{}_{22}$) which shows the chaotic behavior for the given parameters.

The phase curve of the second slave system: Rössler time-delayed chaotic systems ) $x_{31}-x_{33},x_{32}-x_{33},x_{31}-x_{32})$ representing the chaotic behavior for the given parameters.

## 5. Discussion

The presented results reveal good performance of the proposed method in estimating parameters, delays, as well as disturbance bounds and uncertainties. This in turn leads to an acceptable performance of the overall system in synchronization and convergence of errors to zero.

In the rest of the paper, the proposed method was evaluated in secure communication using chaotic masking. To evaluate this, five sinusoid signals were added as messages to the master system’s signal, independently. The decryption and recovering of each message was done by synchronization of the master system with two slave systems. The message signals added to the master system are given below:

(63) $$S_{1}\left(t\right)\;=\;sin\left(0.7t\right)+2cos\left(5t\right)+4sin\left(\pi t\right)\;S_{2}\left(t\right)\;=\;sin\left(0.8t\right)+2cos\left(8t\right)+5sin\left(\pi t\right)\;S_{3}\left(t\right)\;=\;2sin\left(0.85t\right)+5cos\left(5.1t\right)+6sin\left(\pi t\right)\;S_{4}\left(t\right)\;=\;2.5sin\left(0.85t\right)+3.2cos\left(5.16t\right)+5.5sin\left(\pi t\right)\;S_{5}\left(t\right)\;=\;2.5sin\left(0.85t\right)+3.2cos\left(5.16t\right)+4.5sin\left(\pi t\right)$$

**Figure 9:**
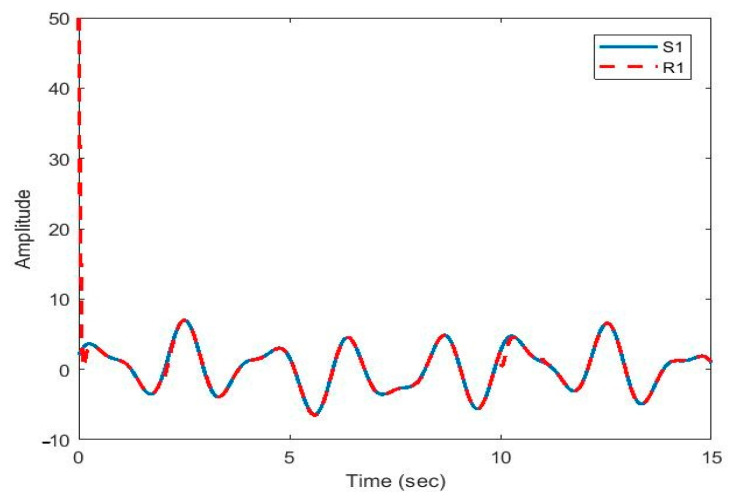
Plot of original signal $\left(S_{1}\right)$ and recovered signal $\left(R_{1}\right)$ obtained using multi-state synchronization and chaotic masking.

**Figure 10:**
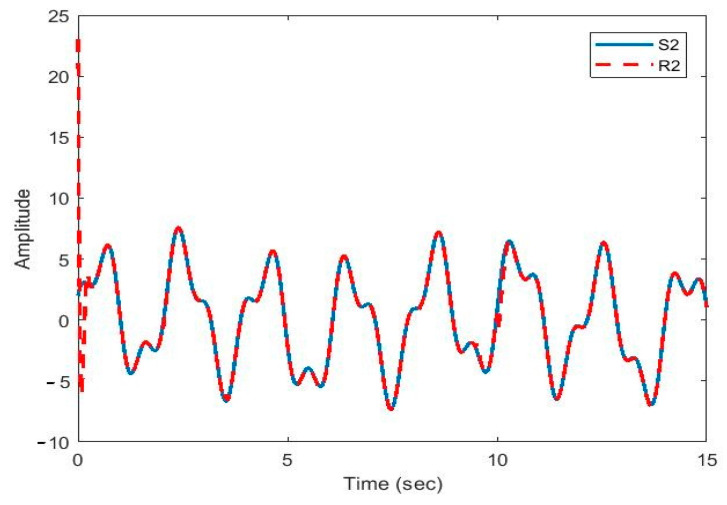
Plot of original signal $\left(S_{2}\right)$ and recovered signal $\left(R_{2}\right)$ obtained using multi-state synchronization and chaotic masking.

**Figure 11:**
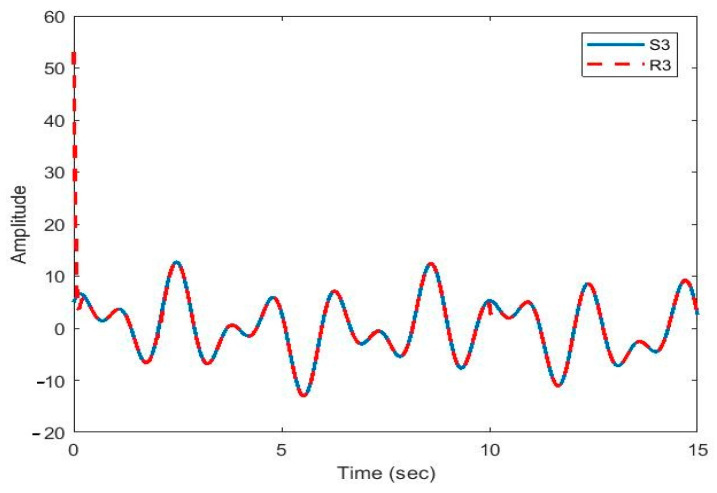
Plot of original signal $\left(S_{3}\right)$ and recovered signal $\left(R_{3}\right)$ obtained using multi-state synchronization and chaotic masking.

**Figure 12:**
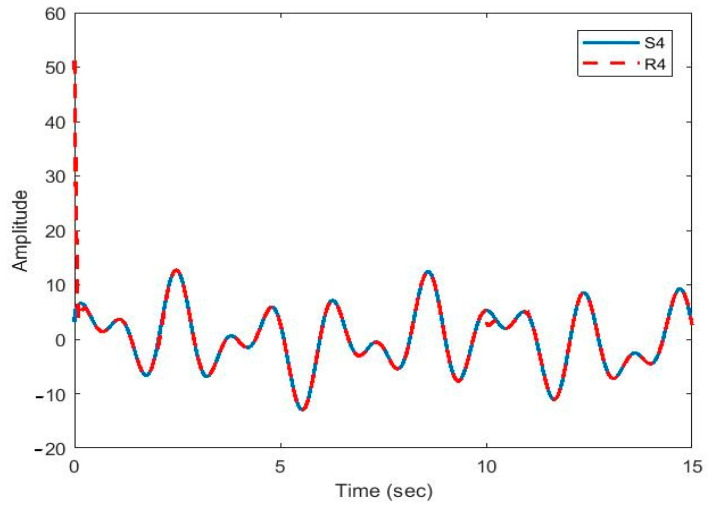
Plot of original signal $\left(S_{4}\right)$ and recovered signal $\left(R_{4}\right)$ obtained using multi-state synchronization and chaotic masking.

**Figure 13:**
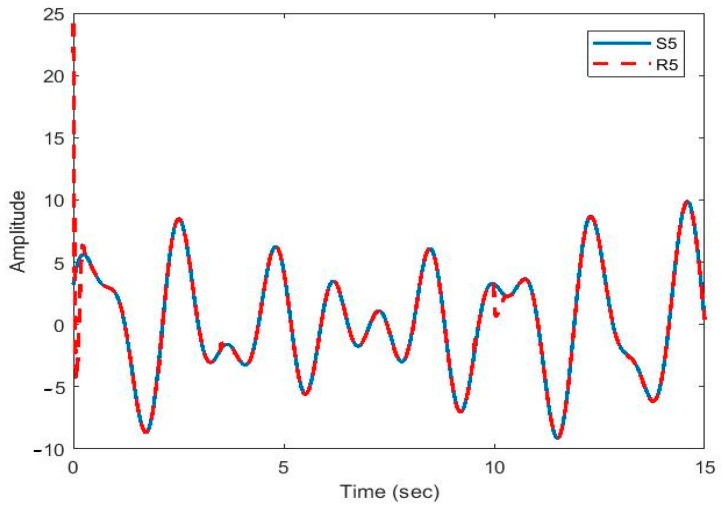
Plot of original signal $\left(S_{5}\right)$ and recovered signal $\left(R_{5}\right)$ obtained using multi-state synchronization and chaotic masking.

After the synchronization, the recovered signals were obtained. In Figure 9, Figure 10, Figure 11, Figure 12 and Figure 13, the original and recovered message signals obtained using multi-state synchronization and chaotic masking are shown.

Figure 9 shows the recovered signal (R_1_) followed the original signal (S_1_) very well. Due to sudden changes in delays and parameters, there was a pick at 10 s, which was dealt with, and after that the recovered signal faithfully followed the original signal again.

Figure 10, Figure 11, Figure 12 and Figure 13 show that the original signal given to the master and received by the slave has been recovered faithfully using our proposed multi-state synchronization method. The advantages of the proposed method are as follows:

Guaranteed closed loop stability in the presence of disturbance, uncertainty, parameter changes, and delays.

1. Accurate estimation of variable time delays and parameters.
2. Specifying the control laws as continuous time functions.
3. Capable of dealing with disturbance and uncertainties with unknown boundaries.
4. Faithful recovery of message signal in secure communication.

The disadvantages of our method are given below:

1. Involves relatively large magnitude of control signal in a few cases (control functions $u_{13},\;u_{23}$).
2. Changes occur solely in a step-like manner; hence, there is a large distance between parameter changes.

In the future, we intend to extend this work by multi-state synchronization with minimization of sum of control efforts and synchronization of chaotic systems with variable parameters. Secure communication can be employed in medical applications as well [41]. Medical data stored in hospitals contain significant information about the patients. In future work, the proposed method for secure communication can be used for medical applications [42,43,44,45,46,47]. Synchronization of chaotic systems is also used in some other applications such as electromagnetism [48] and mechanics [49,50]. As a future work, the chaotic synchronization method of this paper can be developed and implemented for electromagnetic and mechanical applications.

In this paper, we have proposed that, using our multi-state synchronization and chaotic masking method, secure communication can be achieved. In secure communication, encoding (masking) and accurately recovering the message signal is very important. The most important feature in secure communication is the security of the method and complexity of the algorithm, which makes decoding practically impossible or very difficult. In this regard, important points of the proposed method are given below:

(1) Presence of delay and variable parameters in master and slave systems.
(2) Ability to switch to different slave systems.
(3) Presence of undesirable and unwanted factors such as disturbances and uncertainty.

Therefore, our proposed method helps to ensure secure communication. Accurate retrieval of message signal was another prominent feature of this method. It can be noted from our examples that our proposed method is able to recover the message signal faithfully at the receiver. Hence, our proposed approach has two important properties: the ability to mask the data and to recover the message accurately, which can be used in secure communications.

## 6. Conclusions

In this paper, multi-state synchronization of two time-delayed chaotic systems with unknown parameters and delays in the presence of unknown parameters and external disturbance was investigated. The disturbance and uncertainties have unknown bounds, and the master system was non-smooth. To estimate the parameters and time delays, the Lyapunov method was used. This way, convergence of various types of defined errors were guaranteed, and the adaptive rules for estimation of parameters and time delays were determined. Moreover, the update of rules for the bounds of unknown parameters and external disturbance were established. To evaluate the proposed method, the simulations were carried out in a multi-state synchronization setting with time variable delays and parameters. Our results revealed that the proposed controller was good at reducing the synchronization errors to zero with little oscillations. In addition, despite changes such as step and sinusoid, the time delays have been identified and estimated well. Moreover, the errors related to the bounds of unknown parameters and external disturbance converged to zero quickly. The experimental results revealed the capability and flexibility of the proposed method in synchronization of chaotic systems, parameters, and time delay identification in the presence of uncertainty and disturbance. Finally, the capability of the proposed method to recover the message signals in secure communication applications was presented. Due to time variability of the system, the chaotic behavior was more complex, which leads to better protection in secure communication.

## Data Availability

Data sharing not applicable.
